# Cordycepin, a Characteristic Bioactive Constituent in *Cordyceps militaris*, Ameliorates Hyperuricemia through URAT1 in Hyperuricemic Mice

**DOI:** 10.3389/fmicb.2018.00058

**Published:** 2018-01-25

**Authors:** Tianqiao Yong, Shaodan Chen, Yizhen Xie, Diling Chen, Jiyan Su, Ou Shuai, Chunwei Jiao, Dan Zuo

**Affiliations:** ^1^State Key Laboratory of Applied Microbiology Southern China, Guangdong Provincial Key Laboratory of Microbial Culture Collection and Application and Guangdong Open Laboratory of Applied Microbiology, Guangdong Institute of Microbiology, Guangzhou, China; ^2^Guangdong Yuewei Edible Fungi Technology Co, Guangzhou, China; ^3^Guangzhou Institutes of Biomedicine and Health, Chinese Academy of Sciences, Guangzhou, China

**Keywords:** cordycepin, hyperuricemia, uric acid transporter 1, molecular docking, molecular dynamics

## Abstract

Recently, we've reported the anti-hyperuricemic effects of *Cordyceps militaris*. As a characteristic compound of *C. militaris*, we hypothesized that cordycepin may play a role in preventing hyperurecimia. Remarkably, cordycepin produced important anti-hyperuricemic actions, decreasing SUA (serum uric acid) to 216, 210, and 203 μmol/L (*P* < 0.01) at 15, 30, and 60 mg/kg in comparison of hyperuricemic control (337 μmol/L), closing to normal control (202 μmol/L). Elisa, RT-PCR and western blot analysis demonstrated that the actions may be attributed to its downregulation of uric acid transporter 1 (URAT1) in kidney. Serum creatinine levels and blood urine nitrogen and liver, kidney, and spleen coefficients demonstrated that cordycepin may not impact liver, renal, and spleen functions. In addition, we used computational molecular simulation to investigate the binding mechanism of cordycepin. Of which, van der Waals interaction dominated the binding. Residues TRP290, ARG17, ALA408, GLY411, and MET147 contributed mainly on nonpolar energy. This provided the theoretical guidance to rationally design and synthesis novel URAT1 inhibitors.

## Introduction

Hyperuricemia is induced by long-term purine metabolic disorders with high prevalence, and diagnosed as high SUA status (serum uric acid; >6.0 mg/dL for female and >6.5 or 7.0 mg/dL for male; Rock et al., 2013; Liu et al., 2015), associating with gout, renal diseases, hypertension, hyperlipidemia, and atherosclerosis (Choi and Curhan, 2007). Allopurinol is prescribed clinically for hyperuricemia as a xanthine oxidase (XOD) inhibitor (Pacher et al., 2006). However, it is denounced due to its renal toxicity and Stevens-Johnson syndrome (Halevy et al., 2008). Another XOD inhibitor, Febuxostat, was reported to be associated with cardiovascular complications (Becker et al., 2010). Uricosurics, especially benzbromarone and probenecid, interact with renal transporters to elevate excretions of uric acid for anti-hyperuricemia (Shin et al., 2011). But they are troubled by the associated adverse effects, such as enhancement of 6-mercaptopurine toxicity, allergic, and hypersensitive reactions, and nephropathy as examples (Harrold, 2013). Thus, the discovery of novel agents of greater safety and effectiveness is highly wanted for hyperuricemia.

**Figure 1:**
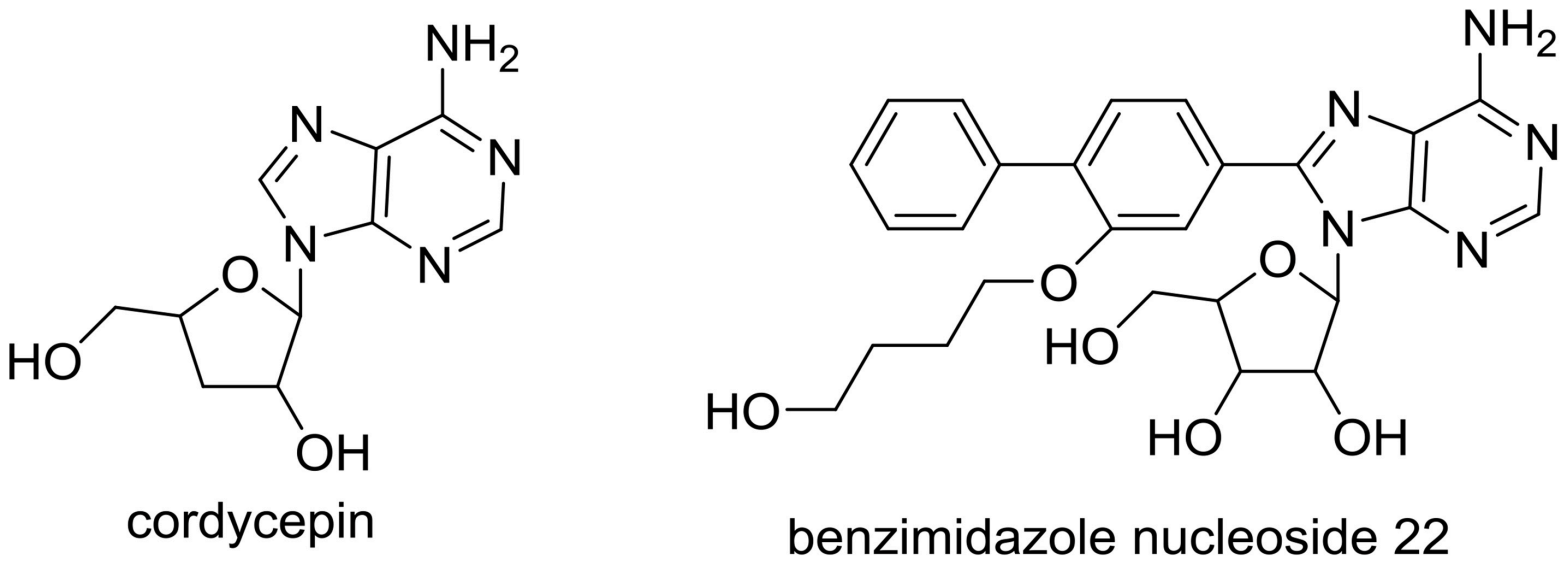
Structure of cordycepin in *C. militaris* and benzimidazole nucleoside 22 for hyperuricemia.

*Cordyceps militaris* has been prescribed against metabolic-associated diseases in traditional Chinese medicine for several centuries (Mizuno, 1999; Ma et al., 2015). We recently reported that *C. militaris* exhibited good anti-hyperuricemic effect in hyperuricmic mice induced by PO and HX with 39–48% ratio reduced in uric acid levels at various doses, approaching the levels of normal mice (Yong et al., 2016). As a characteristic component of *C. militaris*, cordycepin (or 3′-deoxyadenosine, Figure 1), demonstrated various pharmacologic actions, including antifungal (Ahn et al., 2000), antitumor (Zhang et al., 2015), hypoglycemic(Ma et al., 2015), and antiasthmatic effects (Tianzhu et al., 2015). However, its role in *C. militaris* for hyperuricmia has not been included. In addition, adenosine (a cordycepin analog) derivatives (benzimidazole nucleoside 22, Figure 1) have been synthesized and investigated for hyperuricemia (Tatani et al., 2016). Hence, we hypothesized that cordycepin may prevent hyperuricemia.

In this paper, we reported the anti-hyperuricemic effects of cordycepin in hyperuricemic mice. Firstly, SUA and UUA (urine uric acid) were assayed for determine the anti-hyperuricemic effects of cordycepin. Then BUN (blood urine nitrogen) and creatinine were examined. Besides, body weights and organ coefficients were also included. To explore its mechanism, hepatic XOD activities combined with renal GLUT9, URAT1, and OAT1 mRNA and proteins were analyzed. Finally, to learn the binding mechanism of cordycepin, molecular dynamic (MD) simulation was involved here.

## Experimental

### Materials

PO (potassium oxante, 98.0%), HX (hypoxanthine, 99%), allopurinol (98%), and benzbromarone (98%) were brought from Aladdin Reagent Co. (Shanghai, China). Cordycepin (99.5%) was obtained from Target Molecule Corp. (Boston, USA). TRIZOL reagent was offered by Invitrogen Co. (USA). By Nanjing Jian-Cheng Bioengineering Institute (Nanjing, China), Uric acid assay kits were supplied. BUN and creatinine Kits were purchased from Mindray Medical Corp. (Shenzhen, China). From R&D System Inc. (USA), XOD and URAT1 Elisa Kits were obtained.

### Animals, model, and drug administration

All animal experiments were approved by and performed in Guangdong Institute of Microbiology (approved ID: GT-IACUC20170228; Guangzhou, China). From the Guangdong Provincial Medical Laboratory Animal Centre (Guangzhou, China), Kunming mice (20 ± 2 g) were brought. Mice were allowed to have food and water freely for adapting the laboratory conditions before experiment for 1 week. Primarily, mice were separated into normal, hyperuricemic, allopurinol, and benzbromarone controls and cordycepin groups of 15, 30, and 60 mg/kg, respectively.

The protocol reported by us (Yong et al., 2016) was exploited for model establishment with allopurinol and benzbromarone as positive controls. Specifically, mice were dosed with PO (100 mg/kg) intraperitoneally and HX (600 mg/kg) orally simultaneously for models. Meanwhile, normal control mice were dosed with physiological saline (0.9%) at the same time.

For drug administration, animals were treated at 1 h after model building at the frequency at once per day for 7 days. Allopurinol and benzbromarone controls were drugged at 5 and 7.8 mg/kg correspondingly. For cordycepin groups, mice were dosed at a 15, 30, 60, mg/kg. Using physiological saline (0.9%), normal and hyperuricemic controls were treated at the same time.

### Assaying of uric acid, XOD, URAT1, BUN, and creatinine

To test uric acid, BUN, and creatinine according to the manufactures' protocols, serum, and urine were gathered. For XOD activity and URAT1 protein assay by ELISA kits, liver and kidney were collected and tested following the manufactures' protocols.

### Organ coefficient

Liver, kidney, and spleen were washed with saline (0.9%) and sucked with normal filters, followed by weighting. Organ coefficients were computed by dividing the weight of organ by that of corresponding mouse.

### RT-PCR of URAT1, GLUT9, and mOAT1

Total RNA extractions were performed using TRIZOL reagent. After homogenation of kidney tissue, the obtained liquid was added with chloroform and centrifuged, followed by precipitating aqueous phase with volume of isopropanol. After washed by ethanol (75%), the total RNA pellets were suspended using DEPC water. Using RNA (1 μg) together with M-MLV reverse transcriptase, Reverse transcription was conducted. The obtained cDNA was diluted with DNase free water and PCR amplification was performed using primers at appropriate conditions (Table 1) with GAPDH as external standard. Finally, products were quantified by electrophoresis.

**Table 1:** PCR primer sequences and protocols.

**Description**	**Genebank**	**Sense primer (5′−3′)**	**Antisense primer (5′−3′)**	**Product size (bp)**	**T_m_ (°C)**	**Thermal cycle**
GAPDH	NM_008084.2	GTTCCTACCCCCAATGTGTCC	TAGCCCAAGATGCCCTTCAGT	125	60	40
GLUT9	NM_001012363.2	GATGCTCATTGTGGGACGGTT	CTGGACCAAGGCAGGGACAA	241	60	40
URAT1	NM_009203.3	CGCTTCCGACAACCTCAATG	CTTCTGCGCCCAAACCTATCT	254	60	40
OAT1	NM_008766.3	GCCTTGATGGCTGGGTCTATG	AGCCAAAGACATGCCCGAGA	287	60	40

### Western blot analysis

After washing with PBS for three times, kidney cortexes were homogenized with 10-folds of RIPA Lysis Buffer (adding 1 mM PMSF; protease inhibitor) in ice bath. Following bathed on ice for 30 min, the mixtures were centrifuged (12000 g, 10 min). The supernatants were gotten as the total proteins, determined by BCA Protein Assay Kit (Tiangen Biotech Co., China). Before electrophoresis, the total proteins were denatured by incubating for 5 min in boiling water. Samples (5 μg) were separated by 10% SDS-PAGE. And then onto PVDF membrane (Millipore, USA) they were electrophoretically transferred. Non-specific binding sites of obtained membranes were blocked in TBST (Tris-buffered saline with 0.1% Tween-20) mixed with 5% skimmed milk powder. After that, they were then incubated overnight individually with specific antibodies (Table 2) diluted in TBST, consisting of URAT1 (1:2,000), GLUT9 (1:2,000), OAT1 (1:2,000), and GAPDH (1:4,000). After washed for three times with TBST, they were incubated with HRP-conjugated goat anti-rabbit IgG (1:3,000) as the secondary antibody diluted in TBST for 30 min. Consequently, they were washed for three times with TBST and then mixed with ECL (Enhanced Chemiluminescence, Servicebio Co., China). Following that, they were exposed to X-ray film. The contents of target protein were analyzed via densitometry using Alpha Innotech (AlphaEaseShop, USA) and normalized by the respective blotting from GAPDH.

**Table 2:** Antibodies for Western blotting analyses.

**Company**	**Description**	**Catalog number**
ProteinTech Group (Chicago, USA)	Rabbit URAT1 Antibody	14937-1-AP
Novus Biologicals (CO, USA)	Rabbit GLUT9 Antibody	NBP1-05054
Abcam Inc (Cambridge, USA)	OAT1 Antibody	ab135924
Servicebio Co. (Wuhan, China)	GAPDH Antibody	GB13002-m-1

### Statistical analysis

Data were analyzed by ANOVA and showed as mean ± standard error. Significance of difference were at *P* < 0.05 or *P* < 0.01, compared by two-tailed Student's *t*-tests.

### Molecular docking

The molecular docking modeling study was performed with the CDOCKER (Wu et al., 2003). The structure of URAT1 was homology modeled previously (Yong et al., 2016). The binding pocket was determined by alignment with its template. The 3D structures were down from PubChem Compound (ID: 6303) and optimized through energy minimization with the CHARMm force field. Docking calculations were performed using the default parameters.

### MD simulation

Simulation was performed with GROMACS 5.0.4 (Hess et al., 2008; Wennberg et al., 2015). The docked structure of URAT1 with cordycepin was utilized as the start. Geometric optimization and electrostatic potential of cordycepin were calculated at the DFT/6-31G^*^ level. The corresponding topology files of the ligands were generated by the Automated Topology Builder (ATB) server with the charge distribution calculated by DFT/6-31G^*^ level quantum method. The Charmm36 force field was used for URAT1, which were imbedded in POPC membrane and then solvated using the TIP3P water model. To salt and neutralize charges, 0.15 M NaCl were added. Then, the built system was minimized and then equilibrated with cononical (NVT) ensemble. Following that, a further equilibration simulation with the isobaric-isothermic (NPT) ensemble was conducted. The production was carried out in the NPT ensemble. van der Waals (vdW) and long-range electrostatic interactions were ignored beyond 1.2 nm.

### Binding free energy calculation (MM-PBSA)

MM-PBSA (Kumari et al., 2014) was used for binding free energy calculation (ΔG_binding_, Equation 1).

(1) $$\begin{array}{l} \Delta {\text{G}}_{\text{binding}}={\text{G}}_{\text{complex}}-\left({\text{G}}_{\text{receptor}}+{\text{G}}_{\text{ligand}}\right) \end{array}$$

The *G*-value (G_x_) can be represented as Equation (2).

(2) $$\begin{array}{l} {\text{G}}_{\text{x}}={\text{E}}_{\text{mm}}-\text{TS}+{\text{G}}_{\text{solvation}} \end{array}$$

where E_mm_ represents the molecular mechanics energy, TS denotes the entropic contribution, (T, temperature; S, entropy) and G_solvation_ represents the solvation free energy.

Molecular mechanics energy E_mm_ includes the energy of bonded (E_bonded_), electrostatic (E_electrostatic_), and van der Waals (E_vdW_) interactions (Equation 3).

(3) $$\begin{array}{l} {\text{E}}_{\text{mm}}={\text{E}}_{\text{bonded}}+\text{Eelectrostatic}+{\text{E}}_{\text{vdW}} \end{array}$$

G_solvation_ includes electrostatic (G_polar_) and nonelectrostatic (G_apolar_) solvation free energy (Equation 4).

(4) $$\begin{array}{l} {\text{G}}_{\text{solvation}}={\text{G}}_{\text{polar}}+{\text{G}}_{\text{apolar}} \end{array}$$

G_apolar_ was calculated with SASA model (Kumari et al., 2014).

Snapshots extracted from MD trajectory at intervals of 4 ns were used for calculation of MM-PBSA binding free energy. All calculations were performed by use of the g_mmpbsa package developed for GROMACS (Kumari et al., 2014).

## Results

### Anti-hyperuricemic actions of cordycepin through URAT1

**Figure 2:**
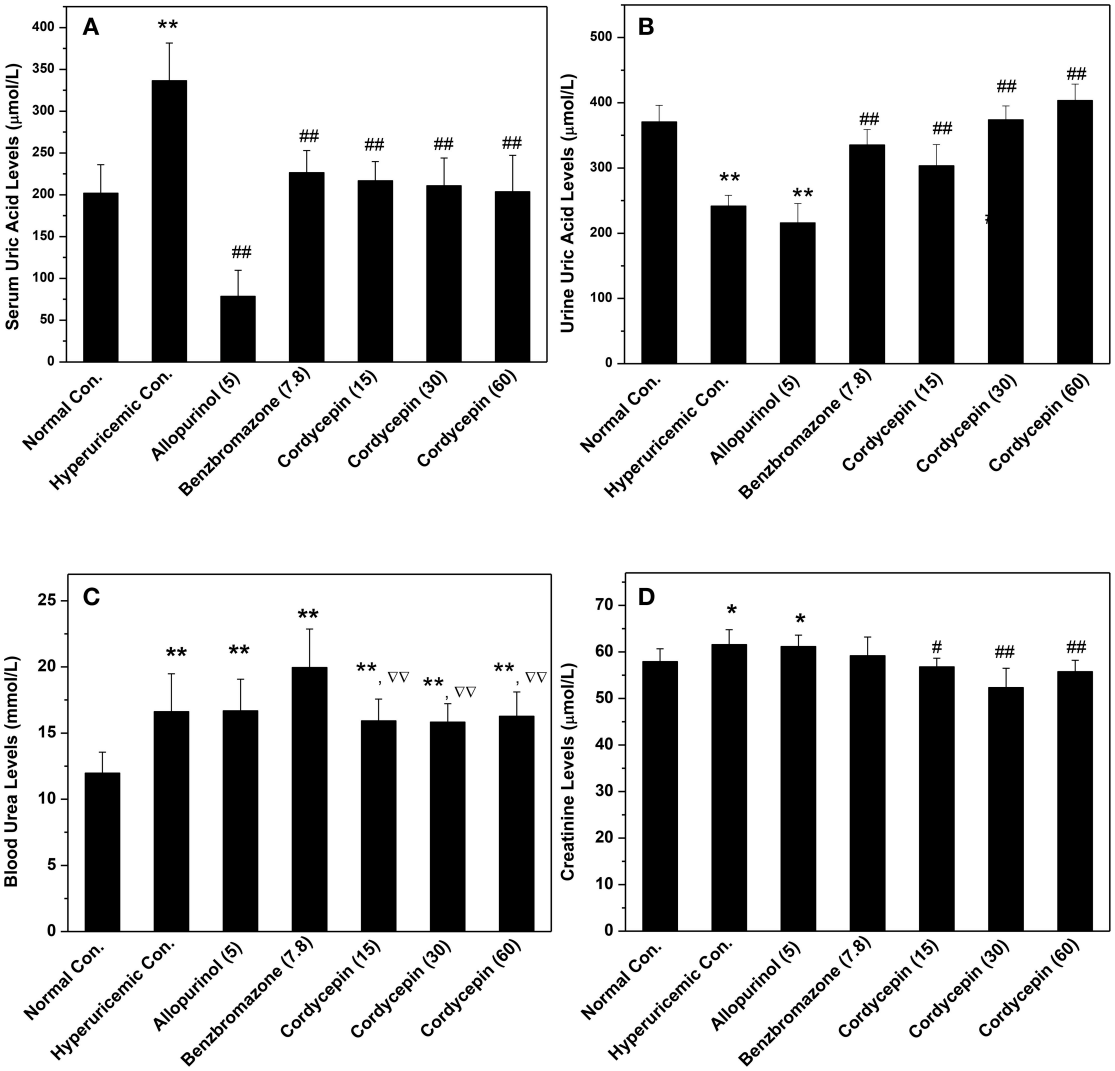
Influence of cordycepin on **(A)** SUA, **(B)** UUA, **(C)** serum creatinine, and **(D)** BUN. ^*^P < 0.05, ^**^P < 0.01 vs. normal control; ^#^*P* < 0.05, ^##^*P* < 0.01 vs. hyperuricemic control; ^ΔΔ^*P* < 0.01 vs. allopurinol control.

As shown in Figure 2A, potassium oxonate and hypoxanthine were further demonstrated to induce hyperuricemia in mice successfully, elevating SUA from normal control (202 μmol/L) to 337 μmol/L for hyperuricemic control (*P* < 0.01). The treatment of allopurinol (5 mg/kg, positive control) in hyperuricemic mice caused significantly declines in SUA to 78 μmol/L (*P* < 0.01), much lower than normal control (*P* < 0.01). For benzbromarone at 7.8 mg/kg, it evoked a significant decline in SUA to 226 μmol/L (*P* < 0.01), nearing normal control. Remarkably, cordycepin produced decreases in SUA to 216, 210, and 203 μmol/L (*P* < 0.01) at 15, 30, and 60 mg/kg correspondingly, closing to normal control.

To elucidate the possibilities that the reductions of SUA by cordycepin were caused by the elevation of renal uric acid excretion, its effects on UUA were detected (Figure 2B). In hyperuricemic control, treatment of PO and HX combindely induced a uric acid excretion decline in mice (241 μmol/L) in comparison with normal control (371 μmol/L, *P* < 0.01). Furthermore, allopurinol induced a further decrease (215 μmol/L, *P* < 0.01), given rise by inhibiting XOD. In contrast, cordycepin at 15, 30, and 60 mg/kg effectively elevated UUA (303, 374, and 403 μmol/L for three doses, *P* < 0.01).

In order to estimate renal functions, BUN and creatinine were examined (Figures 2C,D). BUN for hyperuricemic control (16.61 mmol/L) was higher than that of normal control (11.96 mmol/L, *P* < 0.01), indicating some impairment by amount PO. BUN in allopurinol control (16.67 mmol/L, *P* < 0.01) was 139% of normal control and no significant difference was observed between allopurinol and hyperuricemic controls. Moreover, benzbromarone elevated that to 19.95 mmol/L (*P* < 0.01). In contrast to that, groups of cordycepin demonstrated BUN levels at 15.93, 15.82, and 16.27 mmol/L. Significant increases in serum creatinine were observed in hyperuricemic (61.6 μmol/L, *P* < 0.05) and allopurinol (61.2 μmol/L, *P* < 0.05) controls, contrasting to normal control (57.9 μmol/L, Figure 2D). However, cordycepin decreased that to 56.8, 52.3, and 55.7 μmol/L (*P* < 0.01) in comparison with hyperuricemic control, closing to normal control.

**Figure 3:**
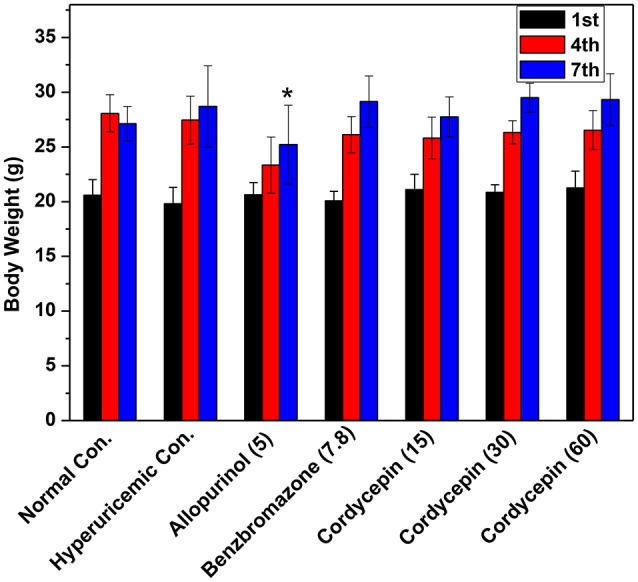
Affections of cordycepin on the body weights of mice detected on the 1st, 4^th^, and 7th days of experiments. ^*^p < 0.05 vs. hyperuricemic control.

Mice body weight data were shown in Figure 3. After acclimatization for 1 week and the establishment of hyperuricemic for another 1 week, normal, hyperuricemic, and benzbromarone controls showed similar body weights. However, allopurinol significantly inhibited the weight growth (*P* < 0.05).

**Figure 4:**
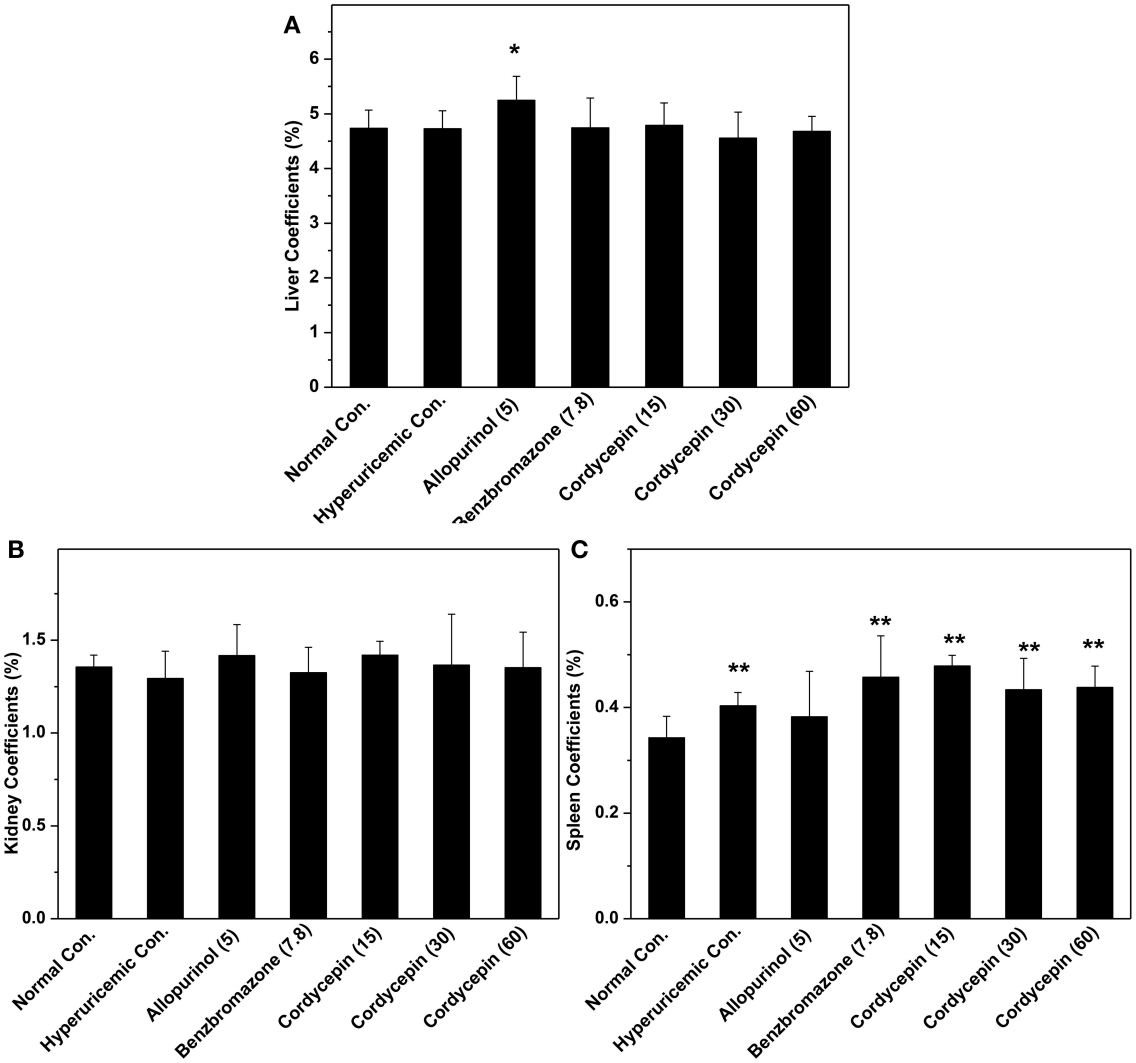
Organ coefficients: **(A)** liver coefficient; **(B)** kidney coefficient; **(C)** spleen coefficient. ^*^P < 0.05, ^**^P < 0.01 vs. hyperuricemic control.

Organ coefficients were recorded for evaluation of effects of cordycepin on inner organ functions (Figure 4). Allopurinol increased liver coefficient in comparison with normal control (*P* < 0.05, Figure 4A). But cordycepin groups showed similarities to normal control. Kidney coefficients for all groups were in neighborhood (Figure 4B). Spleen coefficients for hyperuricemic control (0.40%) were higher than normal control (0.34%, *P* < 0.01, Figure 4C). Allopurinol (0.38%) and benzbromarone (0.45%) controls and cordycepin at three doses (0.47, 0.43, and 0.44%) showed similarity to hyperuricmic control in that.

**Figure 5:**
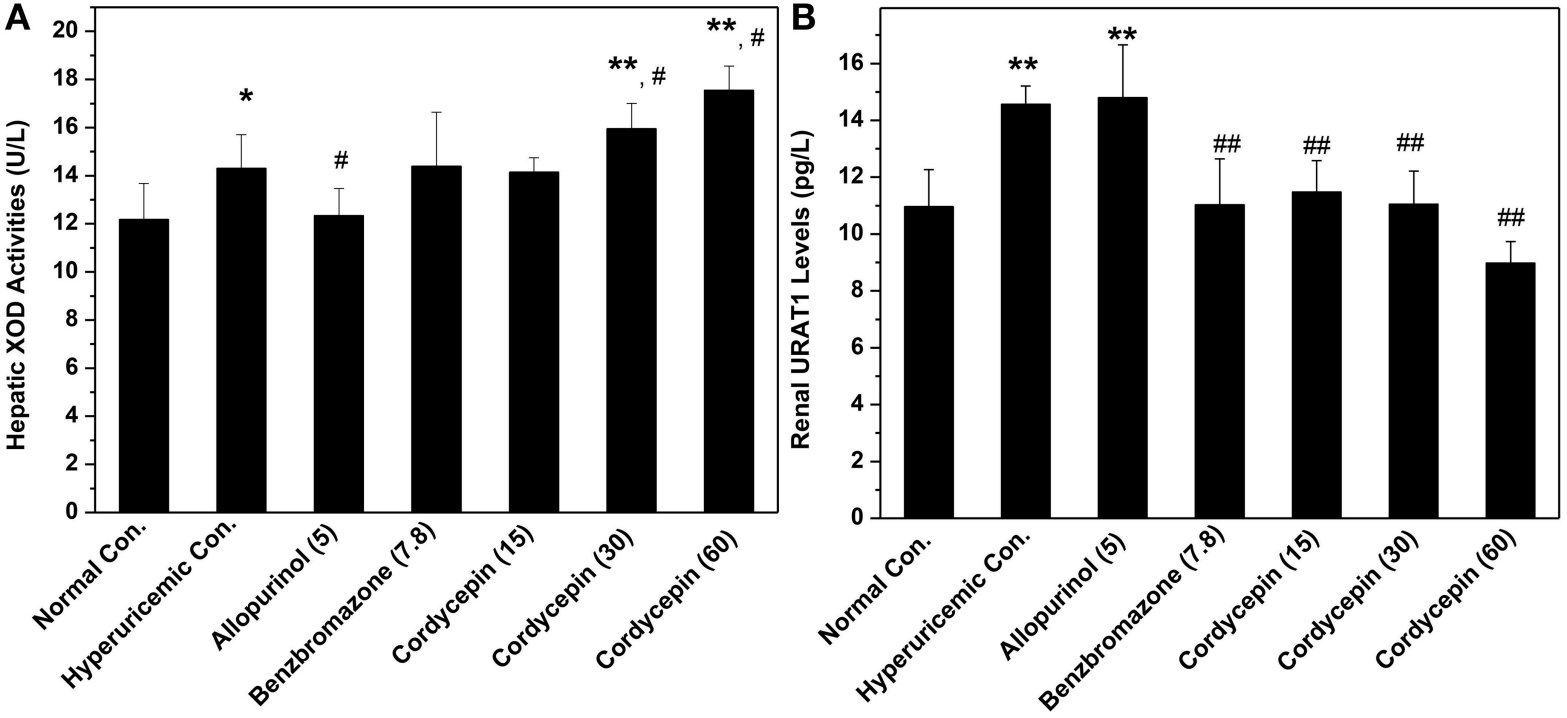
Actions of cordycepin on **(A)** XOD activities and **(B)** URAT1 protein by Elisa. ^*^P < 0.05, ^**^P < 0.01 vs. normal control; ^#^*P* < 0.05, ^##^*P* < 0.01 vs. hyperuricemic control.

XOD activities in hyperuricemic control were ascertained to be 118% fold (14.31 U/L) of normal mice (12.17 U/L, *P* < 0.05, Figure 5A). In contrast to hyperuricemic control, allopurinol control (5 mg/kg) reduced liver XOD activities to 12.33 U/L significantly (*P* < 0.05), closing to that of normal control (12.17 U/L). The none XOD inhibitor, benzbromarone, had not impact on XOD activity (14.39 U/L) comparing with that of hyperuricemic group. Cordycepin at 15, 30, 60 mg/kg showed similar or even enhanced activities to 14.14, 15.94, and 17.54 U/L (*P* > 0.05, *P* < 0.05, and *P* < 0.05) respectively, comparing to hyperuricemic control.

Affections of cordycepin on URAT1 levels of renal organ were examined by Elisa test primarily (Figure 5B). Renal URAT1 of hyperuricemic (14.56 pg/mL) and allopurinol (14.80 pg/mL) groups increased significantly (*P* < 0.01) when compared them with normal control (10.96 pg/mL). The URAT1 levels in hyperuricemic mice treated by cordycepin at various doses were 11.48, 11.05, and 8.98 pg/mL and all was much lower than that of hyperuricemic control (14.56 pg/mL, *P* < 0.01).

**Figure 6:**
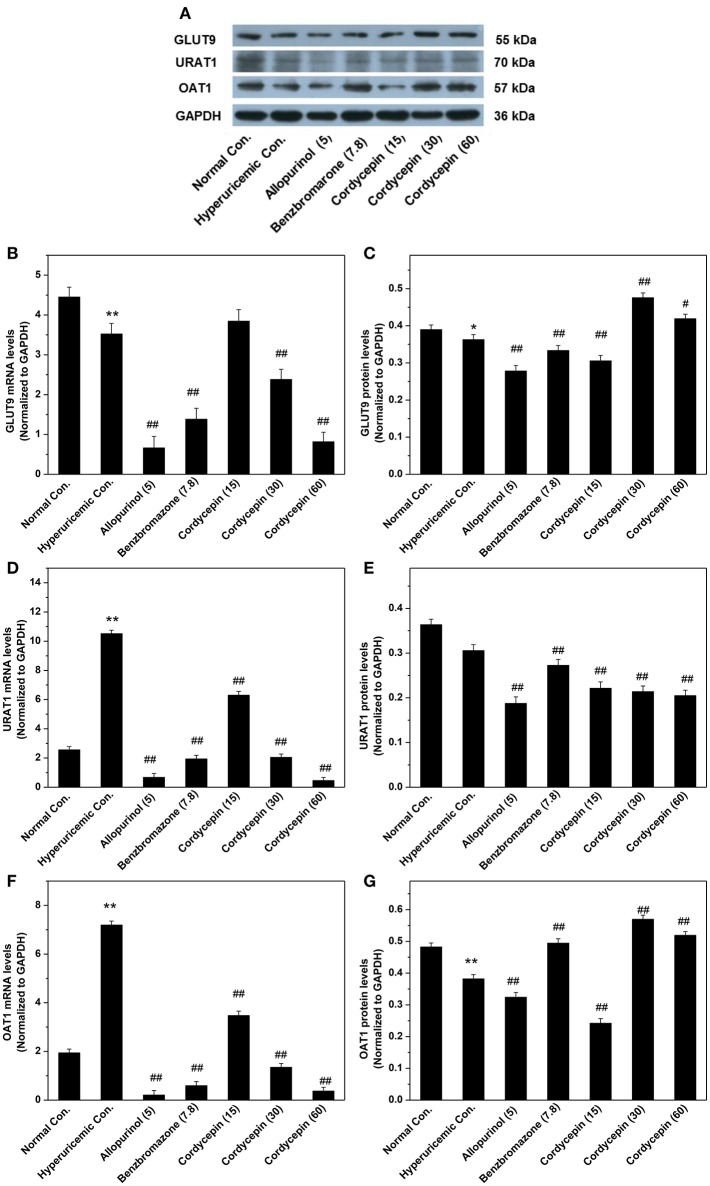
Affections of cordycepin on mRNA and protein of renal GLUT9 **(A–C)**, URAT1 **(A,D,E)**, and OAT1 **(A,F,G)**. ^*^P < 0.05, ^**^P < 0.01 vs. normal control; ^#^*P* < 0.05, ^##^*P* < 0.01 vs. hyperuricemic control.

To further examine the mechanisms of anti-hyperuricemic actions of cordycepin, its affections for GLUT9, URAT1, and OAT1 mRNAs and proteins were examined (Figure 6). For hyperuricemic control, PO down-regulated GLUT9 mRNA (*P* < 0.01) and protein (*P* < 0.05, Figures 6A–C). It also up-regulated URATl and OAT1 mRNA but down-regulated their proteins. Allopurinol and benzbromarone regulated down the GLUT9, URAT1, and OAT1 mRNA expressions and GLUT9 and URAT1 proteins compared with hyperuricemic group. Significantly, 15, 30, and 60 mg/kg cordycepin significantly decreased the URATl mRNA and protein in a dose-dependent pattern. Thirty and sixty milligrams/kilograms cordycepin elevated the GLUT9 and OAT1 protein expressions in kidney, respectively, compared with hyperuricemic control. According to the above, cordycepin may interacts mainly with URAT1 to lower serum uric acid levels. Thus, we tried a computational protocol, including docking, molecular dynamics, and energy calculations, to gather an insight into the binding mode of cordycepin and gain some structural demands on cordycepin inhibitory.

### Interaction of cordycepin for URAT1 by molecular dynamic simulation analysis

**Figure 7:**
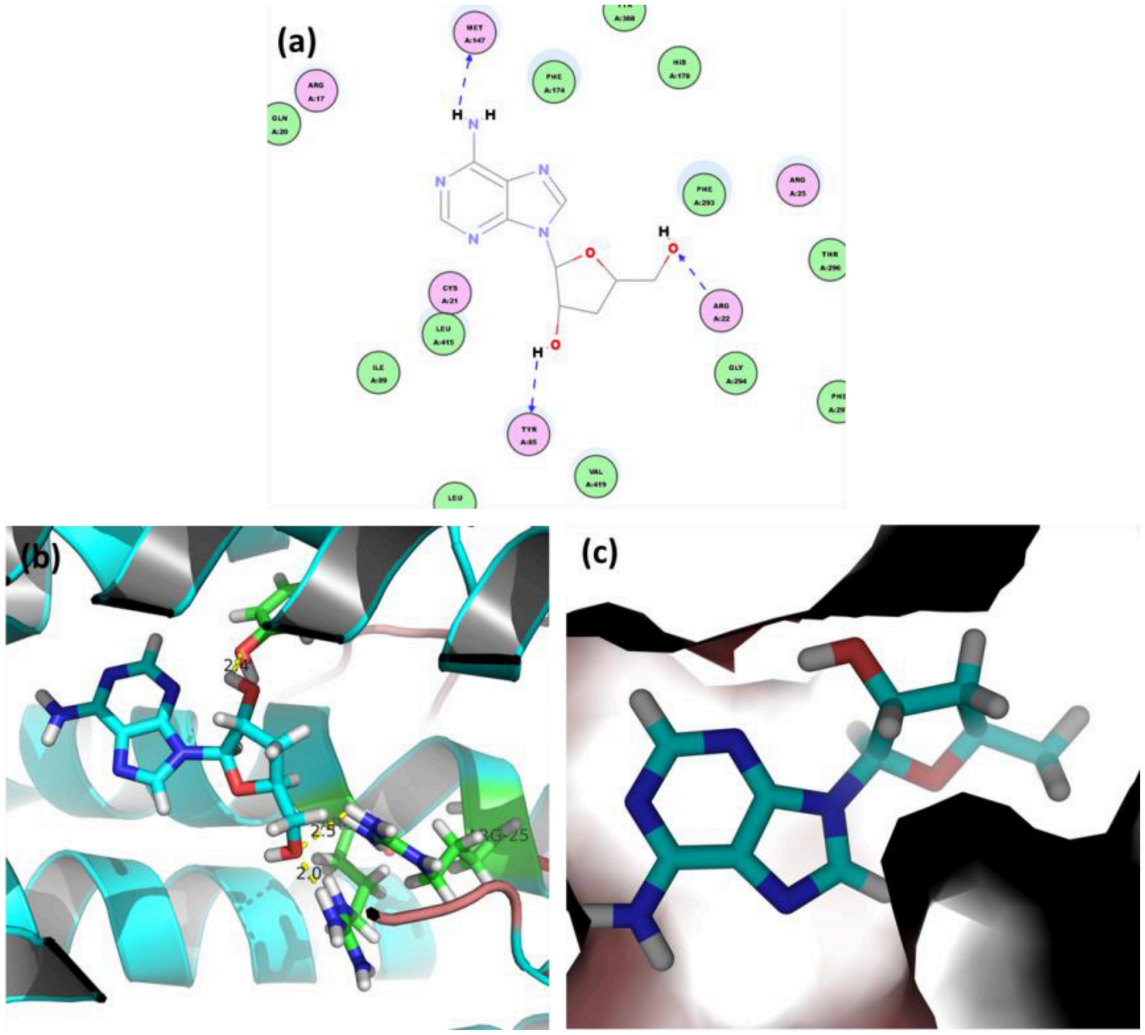
**(a)** 2D view and 3D views **(b,c)** on binding interactions of docked pose.

Figure 7 shows the initial pose of the cordycepin in the active pocket. Figure 7a depicts the two dimensional diagram of the docked cordycepin-protein complex. Apparently, cordycepin bind with the tunnel shaped pocket surrounded by CYS21, ARG22, TYR85, MET147, PHE174, PHE293, and LEU415 via three hydrogen bonds specifically to MET147, ARG22, and TYP85. Wherein, hydrogen donor of the amine on nucleoside of cordycepin bonds with sulfur atom of MET147. Hydrogen and oxygen atoms of two hydroxyl groups of sugar ring bind with oxygen atom of hydroxyl group of TYP85 and hydrogen atom of amine group of ARG22, respectively.

**Figure 8:**
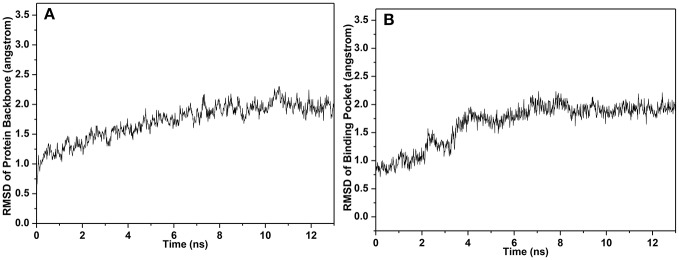
RMSD of **(A)** protein backbone and **(B)** binding pocket.

In simulation, the RMSDs for the protein backbone and the binding pocket were monitored (Figure 8). Accordingly, RMSD for the binding pocket was fluctuant initially and stable after 6 ns. Both RMSDs for protein backbone and binding pocket converged to equilibrium during the last 5 ns. Hence, 500 snapshots extracted from the 5 ns were utilized for structural and energy analysis.

The hydrogen-bond occupancies were computed to examine the binding behaviors of cordycepin (Table 3). Significant hydrogen bond forces were observed for cordycepin with URAT1, where GLY411 as the acceptor for cordycepin occupied up to 11.6% and TRP290 as the donor up to 29.9%.

**Table 3:** Hydrogen bond analysis of MD trajectory.

**Complex**	**Donor**	**Acceptor**	**Distance (Å)**	**Angle (°)**	**Occupancy (%)**
URAT1-cordycepin	N5-H13	411GLY(O)	3.025	25.81	11.6
	290TRP(HE1)	C2-O2	2.893	29.65	29.9

The energy distributions of binding free energy were depicted (Table 4), where the prediction (ΔG_predict_ = −83.324 ± 1.122 kJ/mol) supported the experiment result of binding. Overall, the remarkable nonpolar actions (ΔG_nopolar_ = ΔG_vdw_ + ΔG_sa_; −127.33 kJ/mol) implied the key roles of the hydrophobic residues in pocket for binding; conversely, the polar term (ΔG_polar_ = ΔG_ele_ + ΔG_pb_; 43.99 kJ/mol) was opposite for the integration of URAT1-cordycepin complex. The polar solvation free energies (ΔG_pb_) exhibited an adverse effect on binding.

**Table 4:** Predicted binding free energy and its individual terms (kJ/mol).

**Contribution**	**Cordycepin (kJ/mol)**
ΔG_vdw_	−114.113 ± 0.732
ΔG_ele_	−15.179 ± 0.642
ΔG_pb_	59.170 ± 1.226
ΔG_sa_	−13.222 ± 0.060
ΔG_mm_	−129.29
ΔG_sol_	45.95
ΔG_nopolar_	−127.33
ΔG_polar_	43.99
ΔG_predict_	−83.324 ± 1.122

**Figure 9:**
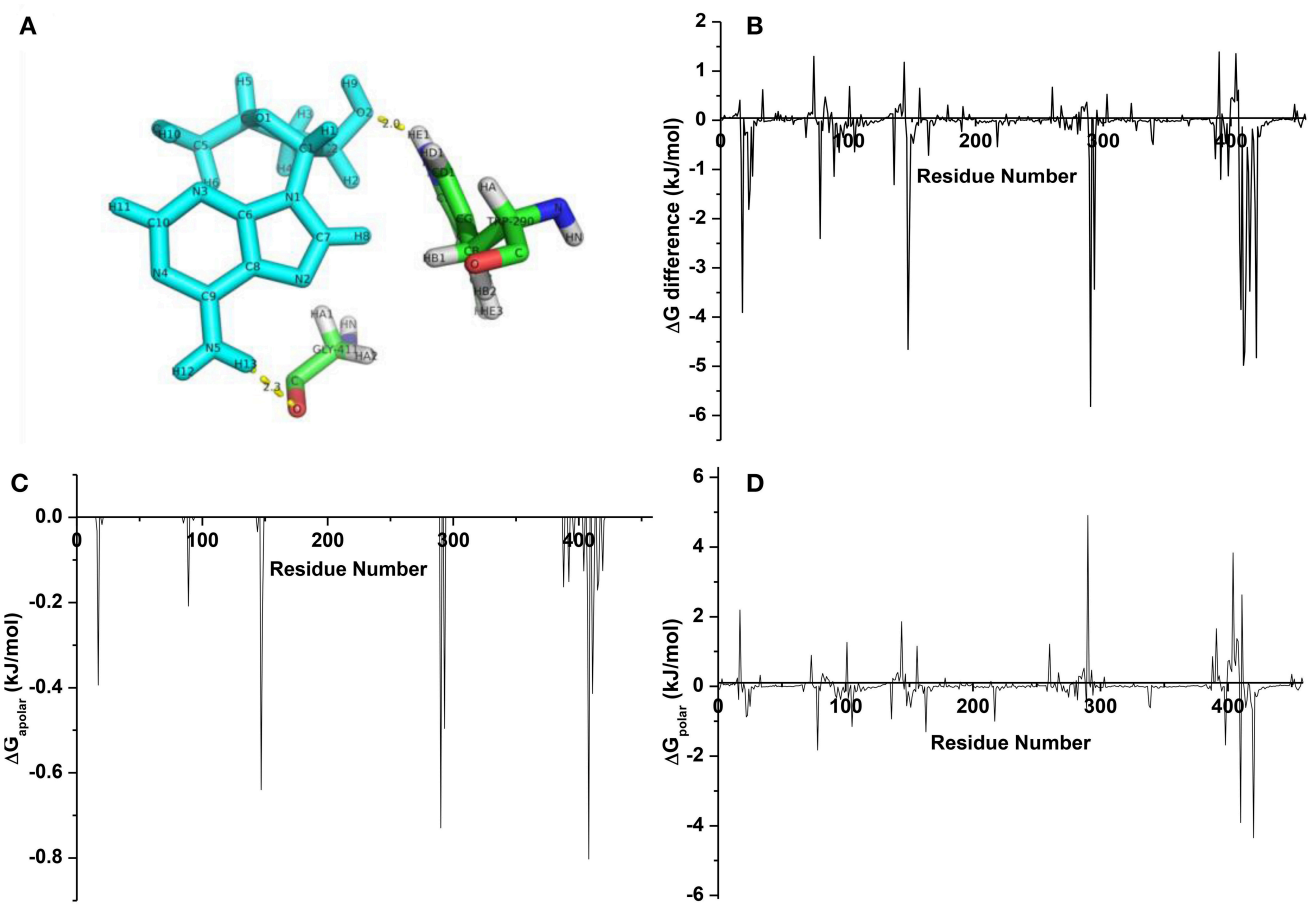
**(A)** The averaged structure for complexes URAT1-cordycepin; **(B)** energy difference of each residue for cordycepin; **(C)** nonpolar interaction spectra; **(D)** polar interaction spectra.

From the averaged structure (Figure 9A), the hydroxymethyl group on the sugar moiety and amine group on the nucleoside part of cordycepin could form HBonds to TRP200 and GLY411, respectively, which made a stable interaction between cordycepin and URAT1. In aim to screen the pivotal residues, energy decomposition was conducted to gain energy contributions of each residue. Obviously, the interaction spectra (Figure 9B) showed that the highlighted favorable residues were TRP290, ARG410, ARG420, GLY411, MET147, ARG17, ALA408, LEU415, PHE293, GLY412, MET148, MET407, and LYS78 at the binding pocket. Consistent with the binding free energy analysis, most of them were hydrophobic, implying for the key role of hydrophobic interaction. Of which, the residues TRP290, ARG17, ALA408, GLY411, and MET147 had important roles in binding, which mainly reflected that the nonpolar interactions (Figure 9C) were driving the complexing process with cordycepin. Besides that, the favorable polar interactions were generated by ARG420 and ARG410, LYS78, and ARG398 (Figure 9D). Thus, the residues ARG420, ARG410, LYS78, and ARG398 contributed strong polar forces to stabilize the URAT1-cordycepin complex, and TRP290, LEU404, GLY411, and ARG17 had an adverse effect on the polar binding.

## Discussion

Cordycepin is a promising compound discovered from *C. militaris* (Cunningham et al., 1950; Kaczka et al., 1964), which has been exploited as a famous traditional herb medicine for kidney and lung disorder therapies for hundreds of years (Kuo et al., 1994). It plays as a characteristic bioactive constituent for this medicinal fugal, whom structure resemble adenosine (Lennon and Suhadolnik, 1976). It benefits cancer therapy in many aspects, such as anti-proliferation (Kuo et al., 1994), anti-migration (Hueng et al., 2017), and inducing apoptosis (Li et al., 2015), in many mechanisms, including affecting lysosomal degradation (Hueng et al., 2017), targeting miR-33b (Zhang et al., 2015), modulating the ERK-JNK signaling pathway (Hwang et al., 2016; Joo et al., 2017), activating p38 MAPK (Baik et al., 2016), and so on. Additionally, it also demonstrated neuroprotective actions (Yuan et al., 2016) through its antioxidant property (Olatunji et al., 2016) and inhibiting CHOP and Bax (Jin et al., 2014). Besides cancer therapy and neuroprotection, metabolic diseases were also focused. For instance, it could prevent hyperlipidemia through activating of AMP-activated protein kinase (Guo et al., 2010) and hyperglycemia through regulation of glucose metabolism (Ma et al., 2015). Recently, we reported the anti-hyperuricemia actions of *C. militaris* through regulation of URAT1 (Yong et al., 2016) and hypothesized that cordycepin may contribute this action. Therefore, we chose cordycepin for hyperuricemia research for the first time.

The hyperuricemic models were built successfully and used for assaying anti-hyperuricemic effects for cordycepin, being consistent with previous report (Yong et al., 2016). Comparison of positive control drug, anti-hyperuricemic efficacy of cordycepin seemed to be similar to benzbromarone, but lower than allopurinol in this study. Obviously, this effective dosage comparison may be irrelevant since further studies should be required. This firstly demonstrates the anti-hyperuricemic actions of cordycepin. SUA is frequently dominated by the renal UUA excretion (Ichida et al., 2012). Cordycepin effectively elevated UUA in hyperuricmic mice, indicating that the anti-hyperuricemic effects of cordycepin may relevant to promoting UUA.

Hyperuricemia causes chronic renal diseases frequently. BUN and creatinine levels are indicators of renal health (Kirtane et al., 2005). In this experiment, PO induced some negative impacts on renal function, but allopurinol had not damaged the renal hardly further. In contrast, cordycepin reversed the elevating of PO on BUN and creatinine, showing some protective impacts on renal function. In other report, its reno-protective effects may be mediated by suppressing Smad2/3 protein and elevating HGF expression (Li et al., 2011).

Analysis of organ weights has traditionally been used for examination of the toxic effects of chemicals for inner organs (Michael et al., 2007). In this study, allopurinol increased liver coefficients, showing some negative impact on liver function. Comparing with it, cordycepin did not show toxicity on liver. Moreover, cordycepin depicted no toxicity on renal function. Finally, it also showed no adverse effects on immune function of spleen.

Because XOD catalyses purines to uric acid (Hille, 1996), alterations of XOD activities may vary serum uric acid concentration directly. In this work, cordycepin did not show suppressive effects on XOD, suggesting that cordycepin may not interact with XOD. In human, excreted uric acids are reabsorbed in kidney. In kidney, URAT1 plays a most powerful role for urate reabsorption (Enomoto et al., 2002). From Elisa result, the URAT1 protein levels in cordycepin groups were lower than that in the hyperuricemic group, showing a dose-dependent pattern. From RT-PCR and western blot, cordycepin administration significantly decreased URAT1 mRNA and protein expression. All above, cordycepin reduced serum uric acid levels through increasing uric acid clearance from kidney by interacting with URAT1.

The effective doses at 15, 30, and 60 mg/kg in mice in this study could be scaled into 85, 171, and 343 mg/day for 60 kg individuals by body surface area (Reagan-Shaw et al., 2008). In other studies, cordycepin did not show toxic effects up to 72 mg/kg for mice (Ma et al., 2015), suggesting daily dosing 411 mg is safe for 60 kg individuals. These imply that the above low doses of cordycepin for hyperuricemia may be safe and effective. But anyway, cautious examination of the efficacy, safety, and toxicology of cordycepin is necessary.

Docking was frequently exploited for building initial poses for MD simulation and investigated binding mode of ligands for targets (Liu et al., 2010). For cordycepin, it docked in the tunnel pocket through three hydrogen bonds, providing evidence for its bioactivity. RMSD of MD simulation showed that the complex of URAT1 with cordycepin is stable and equilibrium in the last 5 ns, confirming that the initial conformation is rational. Hydrogen bond occupancy was analyzed and the high occupied GLY411 and TRP290 should be the key residues for its inhibitory activity.

Cordycepin features a ribofuranose and an adenine nucleoside linked by aβ-N9-glycosidic bond. The main structural features of cordycepin are hydroxyl and amine groups on the nucleoside and ribose sugar, which renders it behaves multi-dental ligand. The interaction spectra depicted that the favorable amino acids of evident contributions were hydrophobic, implying that they form strong van der Waals forces. From the binding free energy decomposition calculation, we could infer that the hydroxymethyl group on the sugar moiety and amine group on the nucleoside part of cordycepin can raise the polar and nonpolar interactions simultaneously to boost the complex forming.

From the above, cordycepin could be a reference for designing novel inhibitors against URAT1, and the most important rules was as following: (1) the residue TRP290 and GLY411 could participate in the stable hydrogen bonds the hydroxymethyl group on the sugar moiety and amine group on the nucleoside part of cordycepin; (2) Based on the important residues TRP290, ARG410, ARG420, GLY411, MET147, ARG17, ALA408, LEU415, PHE293, GLY412, MET148, MET407, and LYS78, the complexes with cordycepin could be stabilized. Hopefully, novel compounds with higher efficacy may be designed and obtained for further research.

In summary, cordycepin demonstrated remarkable anti-hyperuricemic actions in hyperuricemic mice induced by PO and HX. The effect was mediated by down-regulating URAT1. Cordyceps with abundant coydecepin, as a dietary supplement, may be natural remedies for hyperuricemia. Moreover, MD simulation was performed to unveil the binding mechanism of cordycepin and grasp the important structural demands for binding. The energy decomposition showed that nonpolar interaction dominated binding process. Furthermore, energy decomposition of residue unveiled the favorable and none-favorable contributions of residues. In the simulation, nonpolar energy contributions were raised mainly from residues TRP290, ARG17, ALA408, GLY411, and MET147. Our work provided guidance for design URAT1 inhibitors with the favorable bioavailability, safety, and efficacy.

## Author contributions

TY was responsible for the concept and design of the study. TY did the whole experiments of the study and wrote the manuscript. SC, YX, DC, JS, OS, CJ and DZ conducted part of the experiments. All authors participated in the preparation of the manuscript, and have approved the final version.

### Conflict of interest statement

The authors declare that the research was conducted in the absence of any commercial or financial relationships that could be construed as a potential conflict of interest.
